# AI-Based Scientific Manuscript Peer Review: Is It Ready for Adoption?

**DOI:** 10.3390/tomography12060085

**Published:** 2026-06-11

**Authors:** Emilio Quaia

**Affiliations:** Department of Radiology, University of Padova, Via Giustiniani 2, 35128 Padova, Italy; emilio.quaia@unipd.it; Tel.: +39-049-8212375

This Editorial provides insights on artificial intelligence (AI)-based scientific manuscript revision, which could be considered an opportunity to alleviate the reviewer crisis in the field of scientific writing. Most likely, AI-based scientific manuscript revision will be accepted as a further tool in scientific manuscript preparation to assess authors’ own manuscripts, while in the near future, we expect GenAI to have an increased place in scientific manuscript peer review.

In the context of academic publishing, AI-based scientific manuscript revision usually means using AI to improve your own paper’s clarity, structure, grammar, and sometimes argument flow, while preserving the science. This process involves a collaboration where the AI acts as a sophisticated and reliable assistant, while the human author retains ultimate accountability for the scientific accuracy and ethical integrity of the work.

Potential benefits of AI in manuscript writing include AI tools automating tasks such as grammar correction, formatting, and literature review searches, significantly speeding up the manuscript preparation process, the improvement of manuscript readability and grammatical accuracy of papers, which is particularly helpful for non-native English speakers, scientific manuscript adherence to specific journal guidelines, reducing the chance of technical rejection, and data analysis and visual representation of data, enhancing the quality of research papers. Presently, researchers may use AI to self-review their work before it reaches an editor. This includes checking for plagiarism, refining the abstract, and ensuring that the manuscript aligns with the target journal’s guidelines. Journals may use AI to triage manuscripts, select reviewers, or assist external reviewers in identifying technical flaws such as inappropriate statistical tests or missing data.

AI tools for scientific manuscript revision, such as Paperpal, Scite Assistant, and ScholarsReview, automate editing to improve clarity, academic tone, and logical flow while checking for citation accuracy and plagiarism. These platforms, including specialized tools such as q.e.d Science, can identify weak arguments and suggest structural improvements within 30 min, significantly reducing manual revision time.

AI tools (such as Grammarly or Trinka AI) are already used to fix grammar, awkward phrasing, repetition, and journal style and tone. Advanced large language models (LLMs) can suggest reorganizing paragraphs, assess study design methodology, clarifying complex ideas, identifying gaps in the narrative flow, and assess the value of statistical analysis, appropriateness of references, and language and formatting. Tools such as Penelope.ai or AIRA automatically check for adherence to journal-specific formatting, missing sections (e.g., funding statements), and citation consistency. Moreover, AI may facilitate contributions to the biomedical literature (the majority of which is published in English) from non-Anglophone authors, thereby overcoming language barriers. A further possible application of AI tools consists of writing rebuttal letters, point-by-point responses, and revised sections after peer review.

Most major publishers have established clear rules for AI revision since AI cannot be listed as a co-author because it cannot take legal or ethical responsibility for the research, so authors must disclose the use of AI tools in their “methods” section or a dedicated “declaration of generative AI” statement.

Major publishers, along with funding agencies (e.g., NIH, NSF), expressly prohibit uploading unpublished manuscripts into Generative AI (GenAI) tools, as this violates author confidentiality. Most journals strictly prohibit reviewers from entering manuscript content into public AI tools, as this violates the confidentiality of the peer review process. A 2024 review of the top 100 medical journals revealed that 78% of journals provided guidance on AI use, 59% prohibited its use, and 39% allowed its use “if confidentiality is maintained and authorship rights are respected.” Of the journals that provided guidance, 93% prohibited uploading manuscript content to AI, and 32% mandated that AI use be documented in their review [1].

But let us imagine that GenAI-based manuscript revision could be possible for submitted manuscripts. What are the possible advantages? Manuscript reviewers and the accuracy of the review process are fundamental to the quality of a scientific journal. A careful evaluation of the submitted manuscript implies the assessment of its scientific merit and its ranking compared to the existing literature [2]. Nowadays, the role of the reviewer is facing a real crisis since reviewing is a time-consuming process and the increasing pressure to publish may make reviewers less available to dedicate time to assessing the work of their colleagues [2]. Presently, double-blind reviews do not completely reduce gender or affiliation bias or ensure that reviewers disclose conflicts of interest. GenAI-based manuscript revision could be a fantastic resource for these purposes. The present reviewer crisis could be resolved immediately, and reviewer contributions would not be as pitiful for scientific journals, since generative AI represents an endless supply, especially in scientific fields where is very difficult to find competent and dedicated reviewer. Even small journals could provide quick and exhaustive manuscript review with a limited risk of unfair scientific revision. Junior authors will be able to undergo fair revision with a limited and controlled risk of underestimation of their manuscript, while manuscripts from famous scientists or prestigious academic institutions will not be treated with special consideration. In other words, manuscript revision will be impartial, since GenAI can be considered unbiased, transparent, accurate, consistent, confidential, and explainable, since it does not imply human emotions and personal impression. The risks of redundant publication, plagiarism, unethical behavior, and even honorary authorship will be limited [3,4].

What are the possible risks related to scientific manuscript peer review based on GenAI? The risk of abuse is the most serious, since there could be a tendency to over-rely on GenAI [3,4]. A reduction in critical thinking and breaches of confidentiality represent other possible risks [4]. Presently, GenAI is not able to recognize novelty, scientific rigor, relevance, or how the paper contributes to the body of current literature, as human oversight is presently essential in these tasks. Generally, AI-based manuscript revision could provide sentence-level editing, concise academic phrasing, smoother paragraph transitions, thoroughness and consistency, clearer abstracts and cover letters and identify unclear or redundant text. In other words, what still needs human control corresponds to accuracy and insights from scientific research, bias and ethical concerns, the interpretation of results, and the claim to novelty. Peer review is a complex practice, and it is not only about identifying methodological flaws: it also involves assessing the originality and significance of a manuscript, understanding the disciplinary context, evaluating whether author conclusions advance the field, and even making editorial judgments about priority and impact. On the other hand, GenAI may demonstrate hallucinations, a situation when the model generates inaccurate information. Again, human oversight is still essential to verify AI recommendations, especially when they are confusing or non-intuitive [4]. Moreover, LLMs are only as current as their last training, which could lead to outdated knowledge, particularly in fast-evolving fields such as machine learning and AI. If the training data used to train the LLM are biased, this could also be reflected in the AI-enabled review of scientific papers.

However, when used with appropriate human oversight, GenAI is not antithetical to scientific rigor. As GenAI systems continue to mature, it is expected that the hallucination rate will improve compared to today’s models. I predict that GenAI will have more and more space in scientific manuscript revision in the near future, since GenAI could become capable of improving and recognizing novelty, scientific rigor, relevance, or how the paper contributes to the body of current literature. However, most likely, human oversight will remain essential for the foreseeable future, especially in accountability, ethical judgment, disciplinary context, and scientific values as fundamental elements of scientific research integrity, as AI-generated reviews can be superficial or biased or contain hallucinations.

In conclusion, GenAI is likely to play an increasingly important role in scientific manuscript review and revision. Continued advances in LLMs may enhance their ability to evaluate methodological quality, identify inconsistencies, summarize relevant literature, and assist reviewers in assessing manuscripts. However, whether AI will ultimately be capable of reliably judging scientific novelty, significance, and broader scholarly impact remains uncertain. Therefore, human expertise and oversight are expected to remain essential, particularly for evaluating originality, contextual relevance, ethical considerations, and the overall contribution of a manuscript to scientific knowledge.
